# A Restricted Repertoire of De Novo Mutations in *ITPR1* Cause Gillespie Syndrome with Evidence for Dominant-Negative Effect

**DOI:** 10.1016/j.ajhg.2016.03.018

**Published:** 2016-04-21

**Authors:** Meriel McEntagart, Kathleen A. Williamson, Jacqueline K. Rainger, Ann Wheeler, Anne Seawright, Elfride De Baere, Hannah Verdin, L. Therese Bergendahl, Alan Quigley, Joe Rainger, Abhijit Dixit, Ajoy Sarkar, Eduardo López Laso, Rocio Sanchez-Carpintero, Jesus Barrio, Pierre Bitoun, Trine Prescott, Ruth Riise, Shane McKee, Jackie Cook, Lisa McKie, Berten Ceulemans, Françoise Meire, I. Karen Temple, Fabienne Prieur, Jonathan Williams, Penny Clouston, Andrea H. Németh, Siddharth Banka, Hemant Bengani, Mark Handley, Elisabeth Freyer, Allyson Ross, DDD Study, Veronica van Heyningen, Joseph A. Marsh, Frances Elmslie, David R. FitzPatrick

**Affiliations:** 1 Medical Genetics, St George’s University Hospitals NHS Foundation Trust, Cranmer Terrace, London SW17 0RE, UK; 2 MRC Human Genetics Unit, IGMM, University of Edinburgh, Western General Hospital, Edinburgh EH4 2XU, UK; 3 Roslin Institute, University of Edinburgh, Easter Bush, Midlothian EH25 9RG, UK; 4 Service de pédiatrie, CHU Paris Seine-Saint-Denis - Hôpital Jean Verdier Avenue du 14 juillet, 93140 Bondy, France; 5 Department of Medical Genetics, Oslo University Hospital, 0424 Oslo, Norway; 6 Department of Ophthalmology, Innland Hospital, 2418 Elverum, Norway; 7 Northern Ireland Regional Genetics Service, Belfast City Hospital, Belfast BT9 7AB, UK; 8 Clinical Genetics, Nottingham City Hospital, Hucknall Road, Nottingham NG5 1PB, UK; 9 Center for Medical Genetics Ghent (CMGG), Ghent University Hospital, Medical Research Building (MRB), 1st Floor, Room 110.029, De Pintelaan 185, 9000 Ghent, Belgium; 10 Sheffield Clinical Genetics Service, Sheffield Children’s Hospital, Western Bank, Sheffield S10 2TH, UK; 11 Human Development and Health Academic Unit, University Hospital Southampton, Tremona Road, University of Southampton, Southampton SO16 6YD, UK; 12 Service Génétique, Plateau de biologie, CHU Saint Etienne, 42055 Saint Etienne cedex 2, France; 13 Pediatric Neurology Unit, Department of Pediatrics, Reina Sofia University Hospital, Av. Menéndez Pidal s/n, 14004 Córdoba, Spain; 14 Paediatric Neurology Unit, Department of Paediatrics, Clinica Universidad de Navarra, 31008 Pamplona, Spain; 15 DDD Study, Wellcome Trust Sanger Institute, Hinxton, Cambridge CB10 1SA, UK; 16 Manchester Centre for Genomic Medicine, University of Manchester, St. Mary’s Hospital, Oxford Road, Manchester M13 9WL, UK; 17 Oxford University Hospitals NHS Trust, Oxford Medical Genetics Laboratories, The Churchill Hospital, Old Road, Headington, Oxford OX3 7LE, UK; 18 Department of Radiology, Royal Hospital for Sick Children, Edinburgh EH9 1LF, UK; 19 Nuffield Department of Clinical Neurosciences, University of Oxford, Oxford OX3 7LJ, UK; 20 Department of Ophthalmology, Clinica Universidad de Navarra, 31008 Pamplona, Spain; 21 Department of Neurology-Pediatric Neurology, University and University Hospital Antwerp, Antwerp 2650, Belgium; 22 Department of Ophthalmology, Queen Fabiola Children’s University Hospital, 1020 Brussels, Belgium

**Keywords:** iris, aniridia, cerebellar ataxia, cerebellar vermis, cerebellar hypoplasia, ITPR1, calcium, inositol triphosphate, ACTA2

## Abstract

Gillespie syndrome (GS) is characterized by bilateral iris hypoplasia, congenital hypotonia, non-progressive ataxia, and progressive cerebellar atrophy. Trio-based exome sequencing identified de novo mutations in *ITPR1* in three unrelated individuals with GS recruited to the Deciphering Developmental Disorders study. Whole-exome or targeted sequence analysis identified plausible disease-causing *ITPR1* mutations in 10/10 additional GS-affected individuals. These ultra-rare protein-altering variants affected only three residues in ITPR1: Glu2094 missense (one de novo, one co-segregating), Gly2539 missense (five de novo, one inheritance uncertain), and Lys2596 in-frame deletion (four de novo). No clinical or radiological differences were evident between individuals with different mutations. *ITPR1* encodes an inositol 1,4,5-triphosphate-responsive calcium channel. The homo-tetrameric structure has been solved by cryoelectron microscopy. Using estimations of the degree of structural change induced by known recessive- and dominant-negative mutations in other disease-associated multimeric channels, we developed a generalizable computational approach to indicate the likely mutational mechanism. This analysis supports a dominant-negative mechanism for GS variants in ITPR1. In GS-derived lymphoblastoid cell lines (LCLs), the proportion of ITPR1-positive cells using immunofluorescence was significantly higher in mutant than control LCLs, consistent with an abnormality of nuclear calcium signaling feedback control. Super-resolution imaging supports the existence of an ITPR1-lined nucleoplasmic reticulum. Mice with *Itpr1* heterozygous null mutations showed no major iris defects. Purkinje cells of the cerebellum appear to be the most sensitive to impaired ITPR1 function in humans. Iris hypoplasia is likely to result from either complete loss of ITPR1 activity or structure-specific disruption of multimeric interactions.

## Main Text

Ida Mann, in her classic 1925 paper on the development of the iris in human embryos and fetuses,^1^ describes four major morphological stages. From 28 to 49 gestational days (gd), there is formation of the annular irido-hyaloid vessel at the distal rim of the optic cup, coincident with the apposition of the optic fissure and appearance of the lens placode. Between 50 and 77 gd, after the separation of the lens vesicle, the “mesodermal” iris appears as a thin layer distal to the lens, the central regions of which is known as the pupillary membrane. This layer is contiguous with the peri-ocular mesenchyme and the mesenchyme surrounding the hyaloid vessels. From 78 to 84 gd, the ectodermal iris appears as a separate outgrowth from the tip of the optic cup coinciding with the disappearance of the irido-hyaloid vessels. The final stage, from 85 to 175 gd, involves growth of the ectodermal iris, the outer and inner layers of which are contiguous with the future retinal pigment epithelium and the neural retina, respectively. Both layers of the ectodermal iris eventually pigment. The sphincter muscles appear to develop from cells of the distal outer layer supplied by radial vessels from the mesodermal iris. The dilator musculature develops as a thin layer growing radially on the surface on the outer layer of the ectodermal iris.

**Figure 1:**
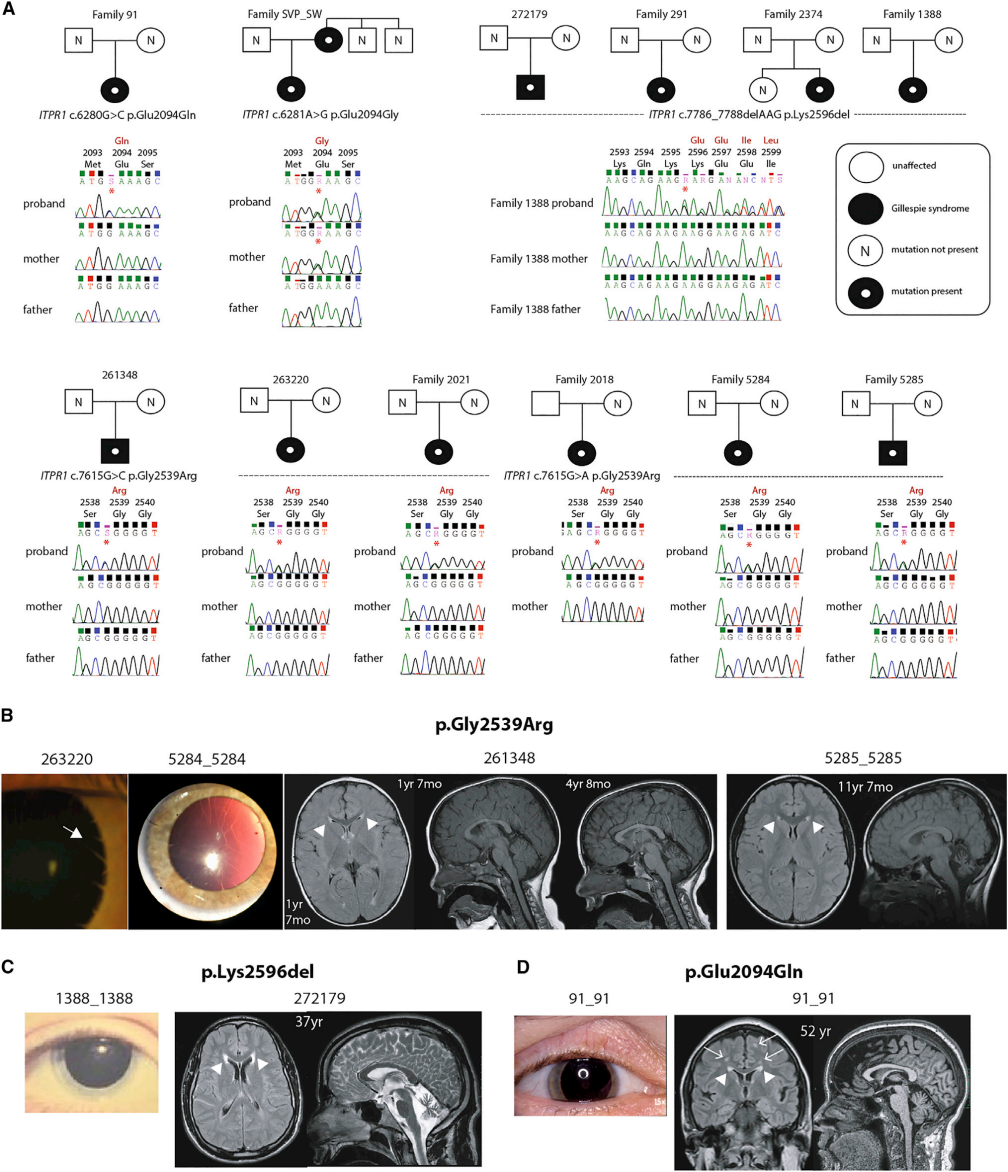
Human Genetic, Ophthalmic, and Radiological Features of Gillespie Syndrome (A) Diagrammatic representation of the Sanger sequencing chromatograms in 12 families in this study with a confident clinical diagnosis of Gillespie syndrome. In 9/12, de novo status of the mutations could be confirmed and in one family (SVP_SW), the mutation was inherited from an affected mother. (B) Left: Image of the right eyes from individuals 263220 and 5284_5284 showing iris hypoplasia and iridolenticular strands (arrowed) typical of Gillespie syndrome. Middle: MR brain imaging of individual 261348 at the age of 1 year 7 months showing minor prominence of the cerebellar folia of the vermis superiorly but by 4 years 8 months progressive cerebellar vermian volume loss and minor prominence of the superior cerebellar folia of both cerebellar hemispheres. Minor periventricular high T2/FLAIR signal adjacent to frontal and occipital horns (white arrowheads). Right: MR brain imaging of individual 5285_5285 aged 11 years 7 months showing moderate vermis and cerebellar hemisphere atrophy, more prominent superiorly and in the vermis with minor increased periventricular white matter T2 signal adjacent to the frontal horns as well as a couple of foci within the frontal lobe white matter bilaterally (white arrowheads). (C) Left: Right eye of individual 1388_1388 showing iris hypoplasia. Right: Individual 272179 at age 37 years. MR brain showing moderate vermis and cerebellar atrophy, worse in the vermis and superiorly. Abnormal periventricular increased T2/FLAIR signal adjacent to the frontal horns (white arrowheads). (D) Right eye of individual 91_91 at age 52 years (de novo c.6280G>C [p.Glu2094Gln]) showing iris hypoplasia with fixed mydriasis. The adjacent MR imaging shows mild cerebellar volume loss (cerebellar hemispheres and vermis), more so superiorly. There is periventricular increased T2/FLAIR signal, most notably adjacent to the frontal horns with multiple foci of white matter increased T2/FLAIR signal elsewhere in the white matter mainly of the centrum semiovale. There is a minor degree of generalized cerebral atrophy. Gyral pattern appears normal.

The best-studied malformation of the iris is complete aniridia (MIM: 106210),^2^ with more than 90% of cases caused by heterozygous loss-of-function (LOF) mutations in the paired- and homeo-domain containing transcription factor *PAX6* (MIM: 607108). *PAX6*-associated aniridia is, however, a pan-ocular disease typified by foveal hypoplasia, cataracts, and progressive corneal opacification in addition to the iris anomaly.^3^ Extraocular disease is rare in *PAX6*-associated aniridia although structural brain anomalies and other sensory impairments have been identified.^4^ Apparently isolated aniridia has also been reported in association with heterozygous LOF mutations in *FOXC1* (MIM: 601090)^5^, ^6^ and *PITX2* (MIM: 601542),^7^ although these loci are more commonly associated with anterior segment dysgenesis (MIM: 602482).^8^ Syndromic forms of aniridia have been described, the best known of which is WAGR (Wilms tumor, aniridia, genital malformations, intellectual disability [retardation] [MIM: 194072]) resulting from a contiguous gene defect encompassing *PAX6* and *WT1* (MIM: 607102).^9^ The other well-known syndromic form of aniridia is Gillespie syndrome (MIM: 206700). Aniridia is, however, an incorrect description of iris malformation in Gillespie syndrome, which is a characteristic form of iris hypoplasia with “scalloping” of the pupillary edge. Gillespie syndrome typically presents as fixed dilated pupils in affected infants. Iridolenticular strands can be seen at regular intervals (Figure 1B) as can other remnants of the pupillary membrane. From the description of the embryology given above, the iris defect in Gillespie syndrome would thus be consistent with a failure of development or maintenance of the sphincter musculature and the associated stroma. The eye in Gillespie syndrome can be further distinguished from *PAX6*-related disease by the absence of foveal hypoplasia and corneal opacification. The key extra-ocular features of Gillespie syndrome are congenital hypotonia, non-progressive cerebellar hypoplasia, and ataxia (Figures 1B–1D) and variable, usually mild, neurocognitive impairment. The inheritance of Gillespie syndrome has been considered heterogeneous with both autosomal-dominant and autosomal-recessive inheritance being postulated on the basis of convincing patterns in individual families.^10^, ^11^ The clinical features of 13 affected individuals with a confident clinical diagnosis of Gillespie syndrome who were used in the molecular studies reported below are summarized in Table 1. We reviewed the available neuroimaging of each case subject, which showed that the cerebellar vermis atrophy is present early and is progressive particularly in the first 5 years of life (Figures 1B–1D). The atrophy mainly affected the superior vermis progressing to involve the superior cerebellar hemispheres more than the inferior aspects. Abnormal periventricular increased T2/FLAIR white matter signal was seen adjacent to the frontal horns on all examinations and older individuals also had scattered foci of increased T2/FLAIR signal in the white matter, mainly frontally. Until now the molecular basis of Gillespie syndrome was not known, with causative mutations in *PAX6*, *FOXC1*, and *PITX2* having been excluded in many reported cases.^12^

Deciphering Developmental Disorders (DDD) is a UK- and Ireland-wide project that aims to use whole-exome sequencing to identify the cause of previously unexplained severe and extreme phenotypes that plausibly have their genesis in embryogenesis or early fetal brain development.^13^ The study has UK Research Ethics Committee approval (10/H0305/83, granted by the Cambridge South REC, and GEN/284/12 granted by the Republic of Ireland REC) with written consent being obtained from all participating families. To date, 13,936 probands have been recruited with DNA samples available in the majority from the affected individual and both parents (trios). Three individuals have been recruited to DDD with a clinical diagnosis of Gillespie syndrome (261348, 263220, 272179; Figure 1A) and these were whole-exome sequenced as part of the first 4,294 trios. The technical and analytical details of the trio exome analysis used in DDD have been previously reported.^14^, ^15^, ^16^ In brief, fragmented genomic DNA was the substrate for targeted pull-down using a custom Agilent SureSelect 55MB Exome Plus and 75-base paired-end sequenced on Illumina HiSeq. Alignment was performed with Burrows-Wheeler Aligner (BWA v.0.59) and realignment around indels with GATK. Putative de novo mutations were identified from exome data with DeNovoGear software.^17^ The functional consequence of each variant was assessed using the most severe consequence from Ensembl Variant Effect Predictor (VEP).^18^ Plausibly pathogenic mutations in known developmental disorders were identified by filtering by gene and allelic requirement using the DDG2P database combined with the minor allele frequencies as described.^16^ Using this approach, each of the Gillespie syndrome case subjects in DDD was found to carry a single plausible pathogenic variant, which was a de novo protein-altering mutation in *ITPR1* (MIM: 147265). Two of these individuals (261348 and 263220) had different heterozygous mutations affecting the same reference base (261348: chr3 g.4856205G>C; 263220: chr3 g.4856205G>A [hg19]), which is predicted to result in an identical change in the open reading frame (p.Gly2539Arg). The latter of these genomic mutations (chr3 g.4856205G>A) is recorded in 1/120,716 (0.000008284) alleles in the ExAC database in an individual of recent African decent, although the inheritance or any associated phenotype of the carrier is not available. Individual 272179 had a heterozygous in-frame deletion of a single codon (chr3 g.4856866_4856868delAAG [p.Lys2596del]). The BAM and VCF files from the first 4,294 trios in the DDD project are available via the European Genome-Phenome Archive (EGA). All residue numbering uses reference sequence GenBank: NP_001161744.1 (Q14643-2; ENSP00000306253.8), which represents ITPR1 isoform 2 with a total of 2,743 amino acids and lacking a 15 amino acid insertion at Asp321. The de novo status of each of these variants was confirmed via an independent sequencing technology (Sanger or Illumina MiSeq). On review of the exome data, no other plausibly pathogenic variant could be identified on the second allele in each of the three DDD case subjects.

After identification of the de novo *ITPR1* mutations in the DDD case subjects, we reviewed whole-exome sequences that had been independently generated on a previously reported^19^, ^20^ mother (SVP) and daughter (SW) with Gillespie syndrome. The exome capture had been performed with the SureSelectXT Human All Exon V5+UTRs kit (Agilent) followed by 150-base paired-end sequencing on a NextSeq 500 (Illumina). The CLC Genomics Workbench v.7.5 was used for read mapping against GRCh37/hg19, followed by duplicate read removal and coverage analysis for all regions enriched with the SureSelect XT exome kit. Approximately 98% of the target regions were covered in both individuals. A read depth of at least 10× was obtained for 80.26% and 90.75% of the SureSelect target regions in both affected individuals, respectively. Finally, quality-based variant calling and annotation was performed and the resulting variant lists were exported for filtering. SVP and SW shared a single, heterozygous, ultra-rare missense mutation (not present in ExAC or 1000 Genomes data) in *ITPR1* (chr3 g.4821268A>G [p.Glu2094Gly]) (Supplemental Data). This study was conducted according to the tenets of Helsinki, and written informed consent was obtained from the participating family.

Eight additional unrelated cases of Gillespie syndrome were identified via the eye malformation cohort held in the MRC Human Genetics Unit (MRC HGU) at the University of Edinburgh, a study approved by the UK Multiregional Ethics Committee (Reference: 06/MRE00/76) with written informed consent obtained from the participating families. Whole-exome sequencing was available on one of these individuals (1388_1388) which, on review, was found to show a heterozygous mutation in *ITPR1* identical to the chr3 g.4856866_4856868delAAG (p.Lys2596del) allele mentioned above (individual 1388_1388 is the same individual as F4:II2 who is described, with the same *ITPR1* mutation, in the accompanying report by Gerber et al.^21^). This mutation was subsequently shown to have occurred as a de novo mutation in this individual. No other plausible disease-causing mutations were identified in *ITPR1* from these exome analyses. Targeted re-sequencing was performed in the seven other individuals with a confident clinical diagnosis of Gillespie syndrome. Six exons of *ITPR1* were selected: coding exons 46 and 52 to 56, which encode the region spanning Glu2094 and the entire calcium ion channel domain, respectively (Table S2). This revealed heterozygous mutations in all seven affected individuals: 4/7, c.7615G>A (p.Gly2539Arg); 2/7, c.7786_7788delAAG (p.Lys2596del); and 1/7, chr3 g.4821267G>C (p.Glu2094Gln) (Figure 1A). In 6/7 of these individuals, the mutation was not present in DNA from the mother and father (all clinically unaffected) and biological relationships were confirmed with highly informative genetic markers suggesting that the mutations had occurred de novo in the affected individual. In 2018_2018, the mutation was not present in the unaffected mother but the father’s DNA sample was not available for analysis. A separate cohort of 173 individuals with non-syndromic aniridia and with no mutation in *PAX6* detected were screened for mutations in *ITPR1* using the targeted resequencing amplicons. No plausible disease-causing mutations were identified, suggesting that *ITPR1* mutations are specific for iris hypoplasia associated with Gillespie syndrome and that this locus does not contribute to other forms of aniridia. Thus, all 13 affected individuals with a clinical diagnosis of Gillespie syndrome that were available to us for study were found to have ultra-rare protein-altering variations affecting only three residues in ITPR1, with at least ten of these mutations having occurred de novo.

**Figure 2:**
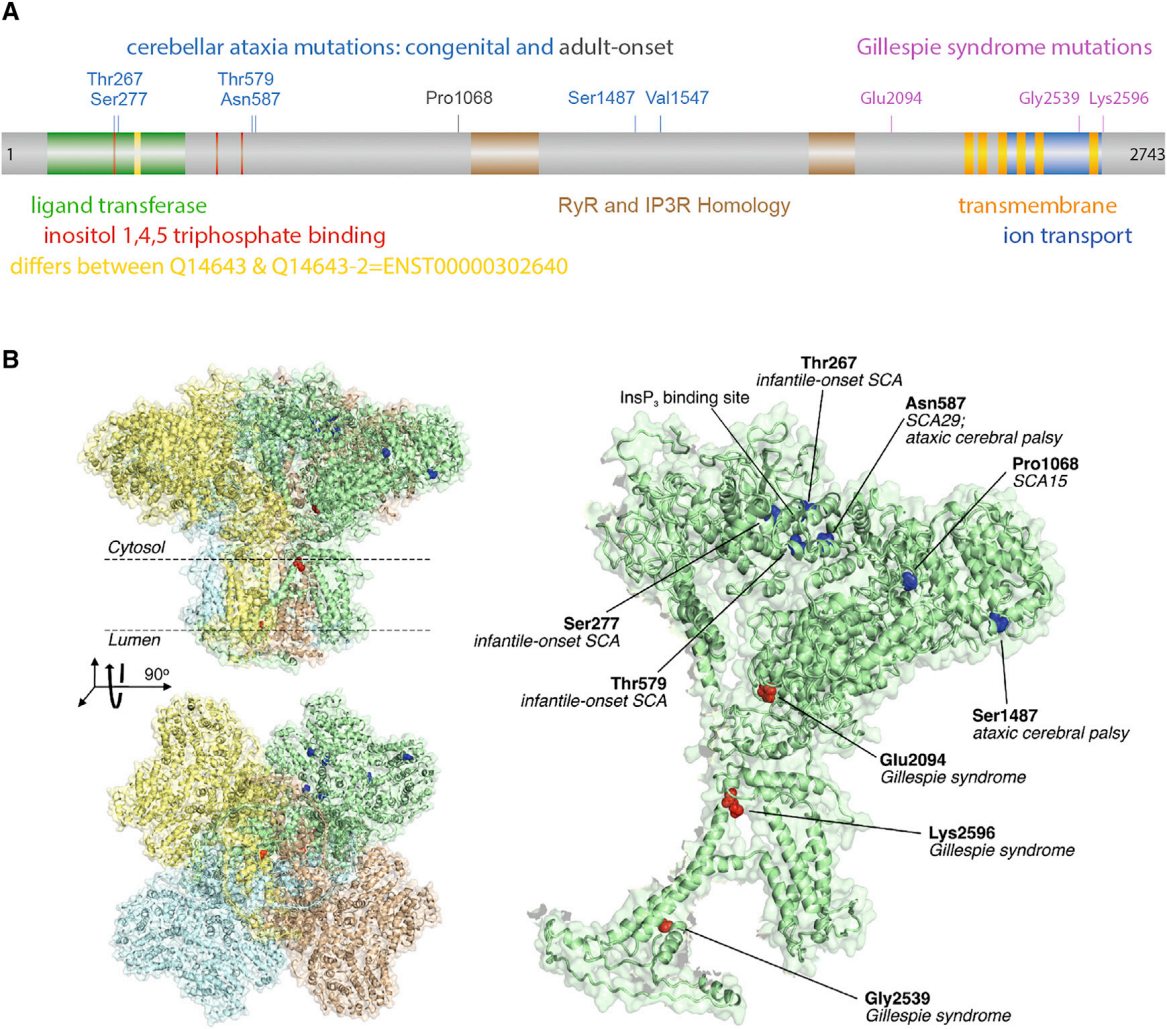
De Novo Mutations Affecting Three Residues in ITPR1 Are the Major Cause of Gillespie Syndrome (A) Linear representation of ITPR1. Amino acid numbering is based on GenBank: NP_001161744.1 (Q14643-2; ENSP00000306253.8), which has 2,743 residues (encoded by the canonical transcript GenBank: NM_001168272.1; ENST00000302640). The colored boxes represent the following domains and features: green, ligand transferase domain; red, inositol 1,4,5-triphosphate binding domain; yellow, 15 amino acid insertion in isoform Q14643-1 (which has 2,758 residues); brown, RyR and IP3R homology domain; orange, intracellular transmembrane domain; blue, calcium ion transport channel. The heterozygous mutations associated with congenital cerebellar ataxia (blue text) mostly cluster toward the N terminus at the ligand transferase and inositol 1,4,5-triphosphate binding domains, whereas those associated with autosomal-dominant Gillespie syndrome (pink text) cluster toward the C terminus at or near the intracellular transmembrane domain and calcium ion transport channel. (B) Structure of the ITPR1 tetramer, left, and monomer, right (derived from PDB: 3JAV). The three mutation sites from this study associated with Gillespie syndrome shown in red, and six sites previously associated with other disorders shown in blue.

*ITPR1* encodes a calcium-release channel that is inositol 1,4,5-trisphosphate (IP_3_) responsive. Heterozygous LOF mutations, mostly deletions encompassing *ITPR1*, have been identified in spinocerebellar ataxia type 15 (SCA15 [MIM: 606658]). SCA15 is characterized by very slowly progressive autosomal-dominant cerebellar ataxia and cerebellar atrophy.^22^, ^23^, ^24^, ^25^, ^26^, ^27^ Haploinsufficiency for *ITPR1* accounted for 2% of dominant ataxia in a screen of a large series of well-characterized families with the age of onset in the affected individuals with *ITPR1* deletions in this series being between 18 and 66 years.^24^ Earlier-onset *ITPR1*-associated cerebellar disease has been reported. In two families with a congenital, non-progressive spinocerebellar ataxia (SCA29 [MIM: 117360]), the disease was found to co-segregate with a different ultra-rare *ITPR1* missense mutation in each family (encoding c.1759A>G [p.Asn587Asp] and c.4639G>A [p.Val1547Met]; these and all subsequent numbering converted to GenBank: NP_001161744.1 [Q14643-2, ENSP00000306253.8] with pathogenicity scores for all variants provided in Table S3).^28^ Another multigeneration family with c.4639G>A (p.Val1547Met) and a mild phenotype have been described.^29^ More recently, de novo missense mutations have been found in infantile onset spinocerbellar ataxia (encoding c.800C>G [p.Thr267Arg], c. 800C>T [p.Thr267Met], c.830G>T [p.Ser277Ile], c.1736C>T [p.Thr579Ile])^30^ and ataxic cerebral palsy (encoding c.1759A>G [p.Asn587Asp], c.4459_4460delinsGA [p.Ser1487Asp]).^31^ In total, eight intragenic mutations, substituting seven residues, have been identified in 12 unrelated cases of cerebellar ataxia, with only one of these cases having an adult-onset phenotype (Figure 2). It is notable that the more severe and earlier-onset *ITPR1*-associated ataxia is caused predominantly by missense variants and that these missense variants are distinct from those associated with Gillespie syndrome. When trying to understand the molecular origins of the dominant phenotype, it is interesting to note that a dominant-negative effect has been described for mutations in several other transmembrane channel genes.^32^, ^33^, ^34^ Thus we can hypothesize that a similar mechanism might account for the effects of the mutations identified here. Given that ITPR1 forms a homotetramer (Figure 2B), then only 1/16 assembled tetramers will contain four wild-type subunits, in the absence of any cotranslational assembly.^35^ If a single variant subunit can block channel function, then 94% of tetramers will be non-functional, thus potentially explaining the dominant phenotype.

We were unaware of any methods for predicting whether protein-altering mutations are likely to show a dominant-negative effect and we speculated that such variants should generally be less structurally perturbing than other LOF pathogenic mutations, because a dominant-negative mechanism requires the complex to at least partially assemble. To address this, we predicted the structural destabilization^36^ of pathogenic missense mutations with a known or likely dominant-negative mechanism from proteins that form transmembrane channels and compared them to recessive mutations from the same proteins or dominant mutations from genes with no known dominant-negative effect (Figure S1). We observe a highly significant difference (p ≤ 0.0015) with the dominant-negative mutations inducing a lesser change in protein stability than the two other groups of mutations.

Next, using the recently determined cryoelectron microscopy structure of the tetrameric ITPR1 protein,^37^ we predicted the effects of the missense mutations identified in this study, as well as the cerebellar ataxia-associated missense mutations mentioned above. All but one of the *ITPR1* mutations are predicted to have mildly destabilizing effects (Table S1). We compared these mutations to a larger set of known dominant-negative mutations in transmembrane channels, recessive mutations in the same transmembrane channels, and other dominant mutations with no known dominant-negative association (Figure S1). We observe that the dominant-negative mutations are significantly less destabilizing than the other groups. The pathogenic missense mutations in *ITPR* were found to be most similar to known dominant-negative mutations using these parameters. Only p.Gly2539Arg is predicted to be strongly destabilizing, although it is still within the range of some of the other known dominant-negative mutations. Additional evidence for the pathogenicity of p.Gly2539Arg comes from its position immediately N-terminal to the ion selectivity filter of the ITPR1 protein.^38^ Indeed, site-directed mutagenesis of Gly2539 to alanine has demonstrated a loss of channel activity in a number of in vitro assays.^39^ Overall, this analysis strongly supports a dominant-negative mechanism for the mutations identified here, as has been observed in other transmembrane channels.

We can also consider how the different *ITPR1* mutations are located with respect to the three-dimensional structure of the complex (Figure 2B). Interestingly, all three residues altered in Gillespie syndrome are located near the center of the channel, within or close to the transmembrane region, whereas all of the non-Gillespie mutations occur away from the center within the cytoplasmic domains. Notably, 4/6 non-Gillespie positions are located at or near the IP_3_ binding site.^37^ The only point mutation associated with adult-onset *ITPR1*-associated ataxia (encoding p.Pro1068Leu) is located relatively near in space to another early-onset mutation, and is also predicted to be only mildly destabilizing, suggesting that it might also be associated with a dominant-negative mechanism, rather than the haploinsufficiency associated with *SCA15* gene deletions.

**Figure 3:**
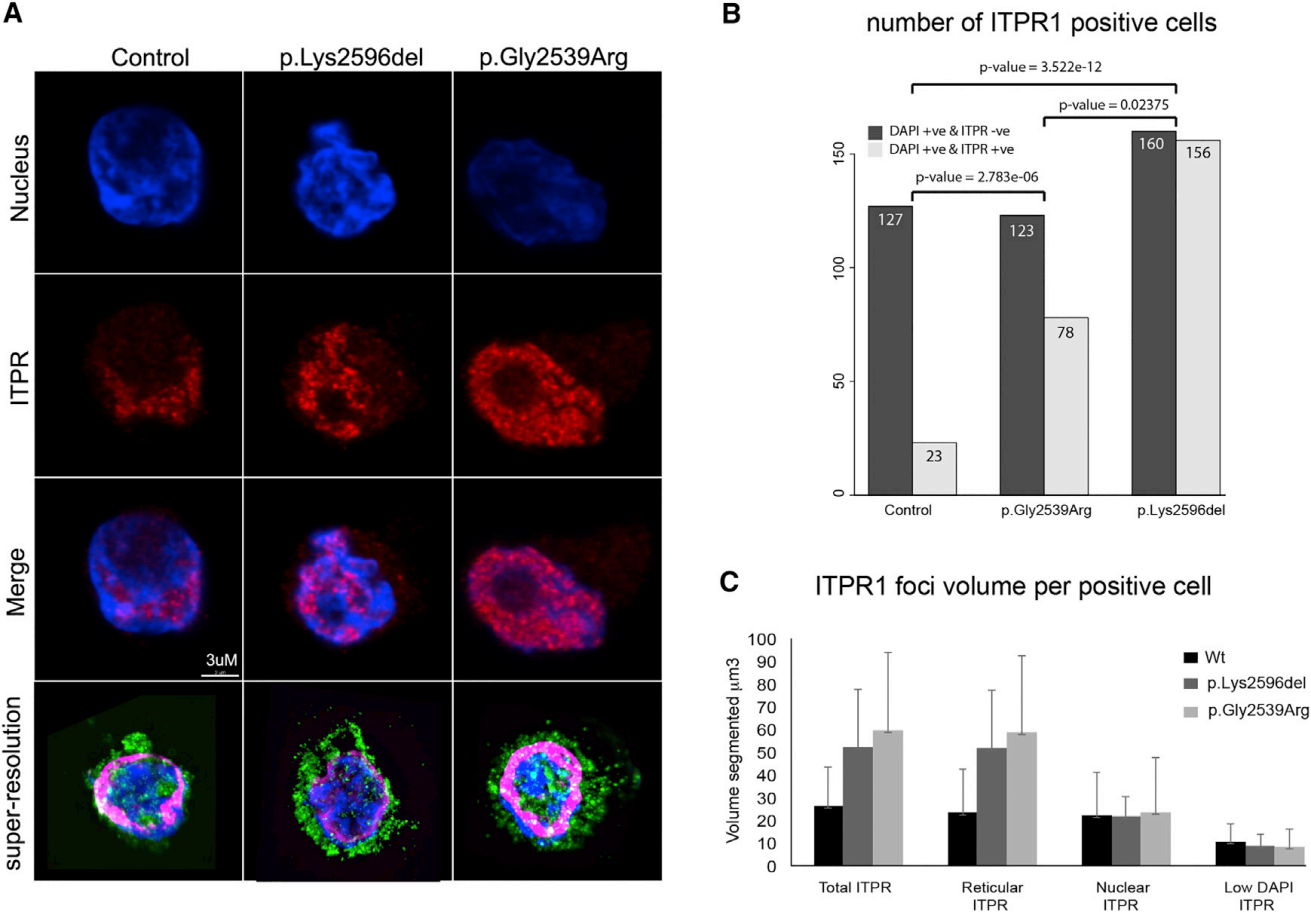
Functional Characterization of ITPR1/Itpr1 Mutations (A) Confocal imaging of lymphoblastoid cell lines (LCLs) showing representative examples from unaffected individuals (control 1 as an exemplar) or individuals with Gillespie syndrome (291_291 and 2018_2018 as exemplars). The top panel shows DAPI-stained nuclei. The panel below shows the punctate staining in the nuclear and perinuclear regions on immunofluorescence staining using an anti-ITPR1 antibody. *ITPR1* mutant cells consistently showed more punctate staining within the nucleus compared to the controls. The third panel shows the merge of the first and second. The fourth panel shows super-resolution SIM imaging of representative LCL nuclei from each of the genotypes, (B) The number of ITPR1-stain-positive cells in LCLs with or without mutations in *ITPR1* were analyzed with ImageJ. Area, shape descriptor, and mean gray value were measured for each cell. In control LCLs, <20% of the DAPI-positive (+ve) cells were also +ve for ITPR1 immunofluorescence. In cells carrying either of the indicated mutations, 30%–50% of the cells were ITPR1 positive. Chi-squared tests of the difference between the mutant and control cells suggest these are very unlikely to be chance observations. (C) Quantitative fluorescence analysis from 3D super-resolution images showing the mean total volume of ITPR1-positive foci for the following compartments within the cell: whole cell, reticular component, whole nucleus, and low-DAPI regions of the nucleus. Multiple individual cells from two independent LCLs derived from affected individuals per genotype were obtained via structured illumination microscopy (SIM). The masking strategy used to obtain these data is outlined in Figure S3. The error bars in this graph represent standard error of the mean. No significant difference was observed between genotypes.

The dominant-negative hypothesis requires the mutant protein to be translated, stable, and correctly targeted. To assess this we used lymphoblastoid cell lines (LCLs) that had been established from five of the affected individuals with Gillespie syndrome. Two of these individuals, 2021_2021 and 2018_2018, carried c.7615G>A (p.Gly2539Arg) and three, 291_291, 2374_2374, and 1388_1388, had c.7786_7788delAAG (p.Lys2596del). Western blot of protein extracted from unsynchronized cultures revealed a variable level of ITPR1 between control and mutant LCL with no obvious difference between the groups (data not shown). Protein localization was assessed using immunofluorescence staining with confocal microscopy or structured illumination microscopy (SIM). As expected, punctate perinuclear staining was seen in both control and mutant cell lines consistent with known localization to the smooth endoplasmic reticulum^40^, ^41^ (Figure 3A). ITPR1 is also known to localize to structures within the nucleus known as the nucleoplasmic reticulum.^42^, ^43^ In the Gillespie syndrome LCLs, the most striking difference compared to control LCLs was a consistently higher proportion of cells that were positive ITPR1 via immunofluorescence (Figure 3B). Using quantitative analysis of super-resolution SIM images, no significant differences could be detected in the number of fluorescence foci or the total volume of the ITPR1-positive regions within the whole cell or the nucleus (Figures 3C and S3). The irregularities in the nuclear outline in the mutant cells might be indicative of an increased number and/or increased size of the nucleoplasmic reticular pores (see Figure 4 in Lui et al.^43^). These changes might reflect failure of a feedback loop caused by a deficit in calcium signaling within the nucleus. However, we were unable to directly assess ITPR1-associated calcium signaling in the LCLs using ATP because no stimulation of calcium signaling was seen in either control or mutant cells (Figure S4).

Heterozygous null, non-mosaic, 16.5 dpc mouse embryos and adult mice were created via CRISPR/Cas9 genome editing methodology (Supplemental Data). These embryos displayed no obvious morphological differences in the early development of the iris compared to their wild-type littermates (Figure S2A). Immunohistochemistry (IHC) of the wild-type mouse embryos revealed no evidence of specific staining of ITPR1 in the developing iris (data not shown). No change in PAX6 levels could be detected between mutant and wild-type embryos (Figure S2A). Two heterozygous null adult mice could be examined at the age of 76 days with wild-type littermate controls (Figure S2B). Although minor defects in the iris were noted in both mice, no major anomalies that would be consistent with the phenotype seen in Gillespie syndrome could be detected. These data suggest that the role of ITPR1 in iris development is either indirect, acting at a later stage of development, or is tolerant of 50% residual channel activity. The latter explanation would be consistent with the lack of an iris phenotype in individuals affected with SCA15 in whom haploinsufficiency for *ITPR1* is the predominant genetic mechanism. Of note, Ca^2+^ has been implicated in development of the eye in both chick and zebrafish, although the source of these ions has been thought to be extracellular (as reviewed in Webb and Miller^44^).

The data presented here provide strong evidence that Gillespie syndrome is a clinically and neuroradiologically distinct disorder that shows locus homogeneity. The cerebellar anomalies in these case subjects are similar to that seen in the SCA29 phenotype. We present evidence based on the predicted effect of mutations on the formation of multimeric channels that suggests that these mutations are likely to be acting by a dominant-negative effect. This protein-structure-based analysis is likely to have wide applicability in the interpretation of mutations, particularly in the important “channelopathy” class of human disease genes.^45^, ^46^, ^47^ The iris hypoplasia, which typifies Gillespie syndrome, might be a consequence of lower level of residual function in ITPR1 (compared to SCA29) but, given that only specific residues are altered, it seems more likely that these mutations disrupt functional interactions that are critical to the formation and/or maintenance of the sphincter pupillae muscle. In this regard it is interesting that mutations in the gene encoding a smooth muscle actin (*ACTA2* [MIM: 102620]) have recently been reported with a very similar iris phenotype.^48^ ITPR1 and ATCA2 might interact in smooth muscle as components of the cGMP kinase signaling complex.^49^

## Figures and Tables

**Table 1:** Summary of the Clinical and Molecular Finding in Individuals with Gillespie Syndrome

**Residue Involved**	**Glu2094**			**Gly2539**						**Lys2596**			
**ID**	**91_91**	**SVP**	**SW**	**261348**	**263220**	**2021_2021**	**2018_2018**	**5284_5284**	**5285_5285**	**272179**	**291_291**	**2374_2374**	**1388_1388**
Genomic mutation hg19	chr3 g.4821267G>C	chr3 g.4821268A>G		chr3 g.4856205G>C	chr3 g.4856205G>A	chr3 g.4856205G>A	chr3 g.4856205G>A	chr3 g.4856205G>A	chr3 g.4856205G>A	chr3 g.4856866_4856868delAAG	chr3 g.4856866_4856868delAAG	chr3 g.4856866_4856868delAAG	chr3 g.4856866_4856868delAAG
Genotype	het	het		het	het	het	het	het	het	het	het	het	het
Mutation type	missense variant	missense variant		missense variant	missense variant	missense variant	missense variant	missense variant	missense variant	inframe deletion	inframe deletion	inframe deletion	inframe deletion
NM_001168272.1; ENST00000302640	c.6280G>C	c.6281A>G		c.7615G>C	c.7615G>A	c.7615G>A	c.7615G>A	c.7615G>A	c.7615G>A	c.7786_7788delAAG	c.7786_7788delAAG	c.7786_7788delAAG	c.7786_7788delAAG
NP_001161744.1; ENSP00000306253.8 consequence	p.Glu2094Gln	p.Glu2094Gly		p.Gly2539Arg	p.Gly2539Ar)	p.Gly2539Ar)	p.Gly2539Ar)	p.Gly2539Ar)	p.Gly2539Ar)	p.Lys2596del	p.Lys2596del	p.Lys2596del	p.Lys2596del
De novo mutation	yes	NK	mat	yes	yes	yes	NK	yes	yes	yes	yes	yes	yes
Sex	female	female	female	male	female	female	female	female	male	male	female	female	female

**Prenatal Growth**													

Gestation	NK	NK	NK	40	40	40	NK	40	NK	37	40	40	37
Birth weight (SD)	NK	NK	“normal”	0.76	1.09	0.99	NK	−1.17	NK	1.25	−1.17	−0.75	0.04

**Postnatal Growth**													

Age (year)	55	34	13	7.19	14.62	28	NK	3.4	12	36.95	10	16	19.75
Height_SD	0.53	NK	NK	−0.38	NK	NK	NK	−3.12	NK	NK	−3	−4.2	1
Weight_SD	−2.31	NK	NK	0.19	NK	NK	NK	−1.7	NK	NK	−2	NK	1.8
OFC_SD	0.35	NK	NK	−0.89	60.7	NK	NK	−0.58	NK	2.39	NK	NK	2

**Neurology and Development**													

Sat independently	late	NK	NK	2 years	2–2.5 years	13 months	NK	9 months	NK	late	18 months	3 years	30 months
Walked independently	8–9 years	NK	NK	10 years	not yet achieved	>6 years	NK	not yet achieved	NK	10 years	not yet achieved	>10 years	7 years
Speech delay	yes	NK	NK	severe	yes	moderate	NK	NK	NK	moderate-severe	yes	yes	yes
Intellectual disability	mild to moderate	learning difficulties	mild	learning difficulties	mild	mild to moderate	NK	mild	moderate	mild-moderate	global delay	global delay	mild
Hypotonia	NK	NK	yes	no	yes	yes	NK	yes	yes	yes	yes	yes	yes, severe
Ataxia	yes	yes	yes	yes	yes	yes	yes	yes	yes	yes	yes	yes	yes, severe
Cerebellar hypoplasia/atrophy	yes	yes	yes	yes	yes	yes	NK	yes	yes	yes	yes	yes	yes

**Ophthalmology**													

Bilateral iris hypoplasia	yes	yes	yes	yes	yes	yes	yes	yes	yes	yes	yes	yes	yes
Foveal hypoplasia	no	NK	NK	NK	yes	NK	NK	NK	NK	no	NK	no	no
Visual impairment	mild	NK	NK	NK	mild	NK	NK	mild	NK	NK	mild	moderate	NK
Negative *PAX6* screen	yes	exome	exome	yes	yes	yes	yes	no	no	yes	yes	yes	yes
Clinical Diagnosis of Gillespie syndrome	yes	yes	yes	yes	yes	yes	yes	yes	yes	yes	yes	yes	yes

**Other Features**													

Other clinical features	gastroesophageal reflux, depression	none	none	gastresophageal reflux	scoliosis, gall stones	none	none	patent foramen ovale and a mild pulmonary valve stenosis	none	scoliosis, macrocephaly, small ears	none	frontal bossing	slight facial dysmorphism

Abbreviations are as follows: NK, not known; Het, heterozygous variant; SD, standard deviation; OFC, occipito-frontal circumference.

## Web Resources

Align GVGD (February 8, 2016), http://agvgd.iarc.fr/agvgd_input.php
DECIPHER, http://decipher.sanger.ac.uk/
Deciphering Developmental Disorders, http://www.ddduk.org/
Ensembl Genome Browser, http://www.ensembl.org/index.html
European Genome-phenome Archive (EGA), https://www.ebi.ac.uk/ega
ExAC Browser, http://exac.broadinstitute.org/
MutationTaster (February 8, 2016), http://www.mutationtaster.org/
NCBI, http://www.ncbi.nlm.nih.gov/
OMIM, http://www.omim.org/
PolyPhen-2 (February 8, 2016), http://genetics.bwh.harvard.edu/pph2/
RCSB Protein Data Bank, http://www.rcsb.org/pdb/home/home.do
RefSeq, http://www.ncbi.nlm.nih.gov/RefSeq
Variant Effect Predictor (February 8, 2016), http://useast.ensembl.org/Homo_sapiens/Tools/VEP

## Supplemental Data

Document S1. Figures S1–S4 and Tables S1–S3

Document S2. Article plus Supplemental Data
