# Supplementary material for: A Restricted Repertoire of De Novo Mutations in ITPR1 Cause Gillespie Syndrome with Evidence for Dominant-Negative Effect
Source: Am J Hum Genet. 2016 Apr 21;98(5):981–92. doi: 10.1016/j.ajhg.2016.03.018 (PMC4863663; doi:10.1016/j.ajhg.2016.03.018)
Supplement: Document S1. Figures S1–S4 and Tables S1–S3 [file mmc1.pdf]

The American Journal of Human Genetics, Volume *98*

Supplemental Data

A Restricted Repertoire of De Novo Mutations in *ITPR1* Cause Gillespie Syndrome with Evidence for Dominant-Negative Effect

Meriel McEntagart, Kathleen A. Williamson, Jacqueline K. Rainger, Ann Wheeler, Anne Seawright, Elfride De Baere, Hannah Verdin, L. Therese Bergendahl, Alan Quigley, Joe Rainger, Abhijit Dixit, Ajoy Sarkar, Eduardo López Laso, Rocio Sanchez-Carpintero, Jesus Barrio, Pierre Bitoun, Trine Prescott, Ruth Riise, Shane McKee, Jackie Cook, Lisa McKie, Berten Ceulemans, Françoise Meire, I. Karen Temple, Fabienne Prieur, Jonathan Williams, Penny Clouston, Andrea H. Németh, Siddharth Banka, Hemant Bengani, Mark Handley, Elisabeth Freyer, Allyson Ross, Veronica van Heyningen, Joseph A. Marsh, Frances Elmslie, David R. FitzPatrick, and DDD Study

Structural consequences of protein altering mutations

Figure S1

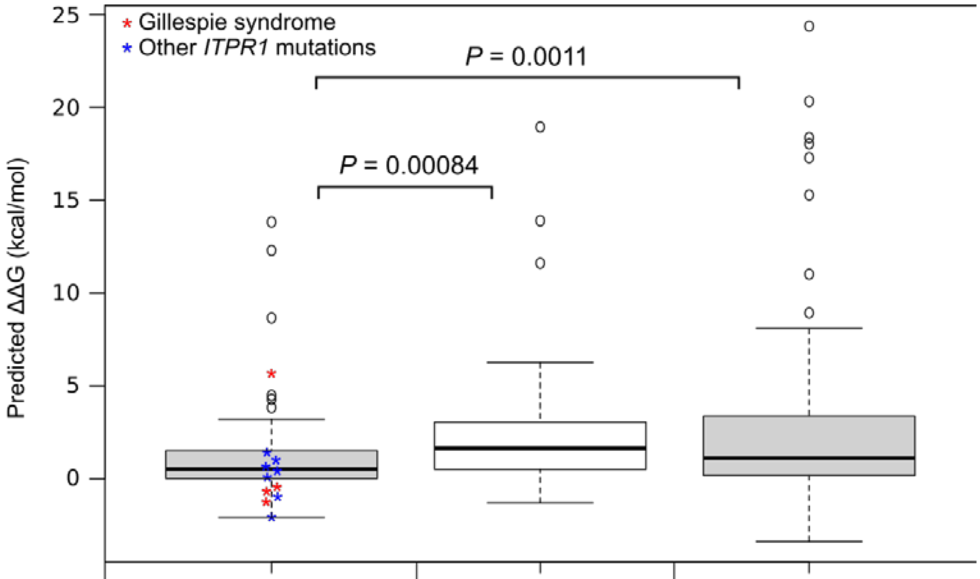

Probability that 11 random mutations have mean ΔΔG lower than seen for *ITPR1*:	Dominant-negative mutations in transmembrane channel genes (*n* = 84)	Recessive mutations in the same set of transembrane channel genes (*n* = 40)	Dominant mutations in genes with no known dominant-negative association (*n* = 163)
	0.080	0.00028	0.0033

Figure S1: Comparison of the predicted changes in protein stability (ΔΔG) for different types of point mutations.

We mapped all human missense mutations annotated as pathogenic in Ensembl Variation 81 against chains with >50% sequence identity in the Protein Data Bank (PDB) as of 2015-10-21. Dominant, recessive and dominant-negative classifications came from Ensembl, OMIM and (ref.1). Dominant mutations from genes with a known dominant-negative effect were included in the dominant-negative group. We used FoldX to predict the change in protein subunit stability (ΔΔG) for each mutation. For mutations that mapped to multiple different structures, we used the average ΔΔG values. Boxes represent quartile distributions and whiskers extend up to 1.5x the interquartile range, with open circles representing outliers. Values for the *ITPR1* mutations (Table S1) are shown with the dominant-negative group, coloured red for mutations identified in this study and blue for those identified previously. *P*-values are calculated with the Wilcoxon rank-sum test and show that dominant-negative mutations in transmembrane channel genes are significantly lower in predicted ΔΔG compared to recessive mutations from the same set of genes, or dominant

mutations from genes with no known dominant-negative association. The values at the bottom represent the probability that 11 mutations randomly selected from each group would have a lower mean $\Delta\Delta G$ than the 11 *ITPR1* mutations considered here, calculated from 10^7 trials. This suggests that the *ITPR1* mutations are most consistent with the dominant-negative mutations and significantly less destabilizing than the recessive or other dominant mutations. If Lys2596del is ignored (since FoldX does not accurately predict the effects of deletions), then the probabilities change to 0.153, 0.0013 and 0.0094 for the three groups.

Figure S2

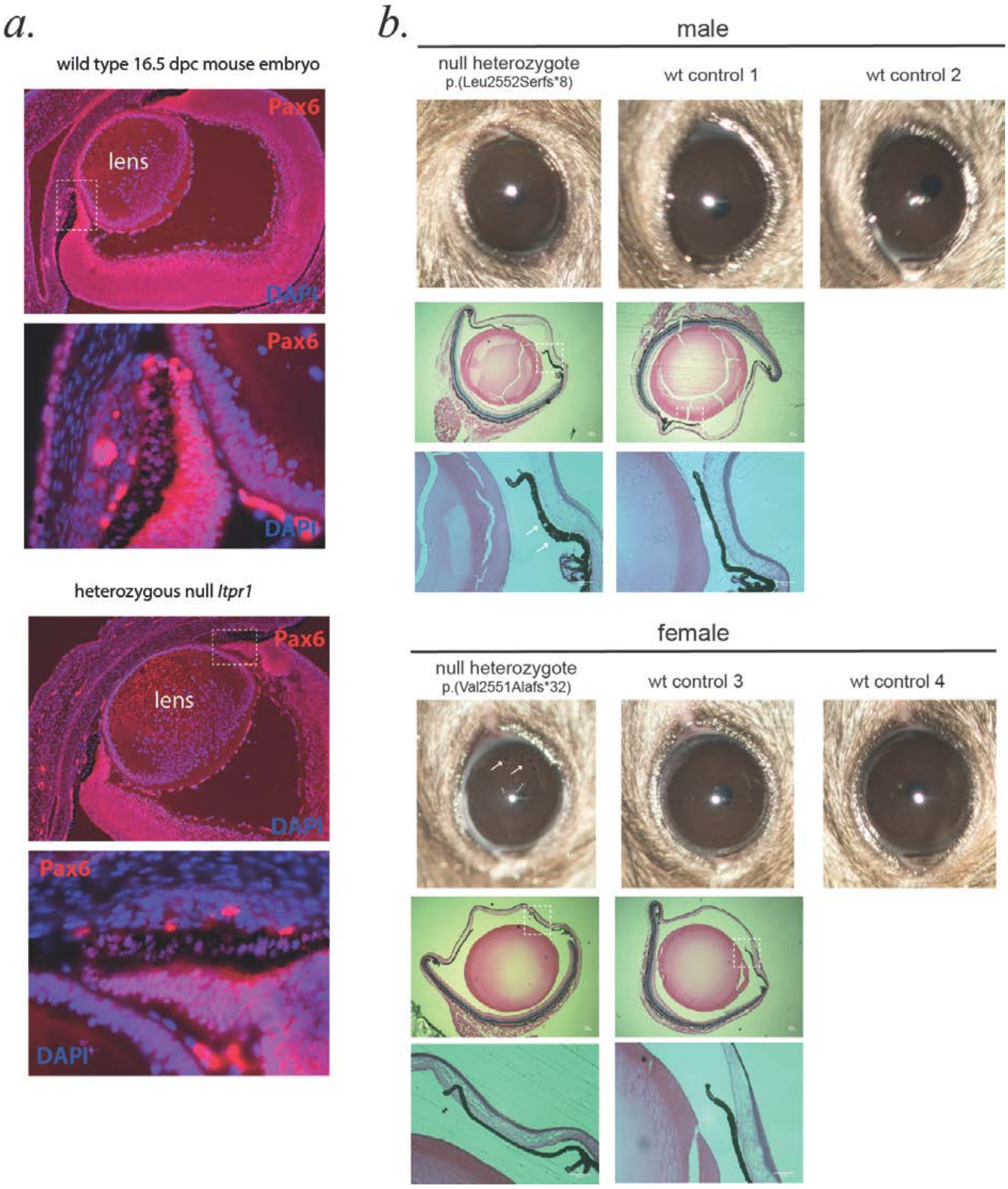

Figure S2: Embryonic and adult phenotype in Itpr1 heterozygous null mice.

A. Saggital sections through the eyes of 16.5 dpc mouse embryos. Representative sections from the wildtype embryos (top, n=3) and the heterozygous Itpr1 null allele littermates (bottom, n=3) generated using CRISPR/Cas9 genomic editing. Guide RNAs were designed

to target coding exon 57 of *Itpr1* (Itpr1_MS_gRNA_1 5'-CACCGCAGTGACGATGCACATGAGC; Itpr1_MS_gRNA _2 5'-CACCGAGGGGTAGGAGACGTGCTC), and their synthesis and microinjection was performed essentially as previously described (ref.2). Immunohistochemical analyses was performed on embryos with concordant genotypes - apparently heterozygous mutation or a wild type allele. The sections were stained using in-house anti-Pax6 monoclonal antibodies, AD1.5.6 and AD2.3.7 using standard immunohistochemical techniques (details available on request) with DAPI counterstain. The top panels are shown at 10x magnification and the boxed regions of the developing iris and ciliary body are shown at 63x magnification below. No difference was discerned between the mutant and wild-type eyes at this stage of development. **B.** Photographs obtained using a slip lamp of the right eye of one male and one female 76-day old mice heterozygous for *Itpr1* null mutations, with two sex-matched littermate controls. In the middle and lower panels are 5x and 10x magnification of 7uM sections of the right eyes of the mutant mice and one control. No major anomalies were seen on microscopy of the iris.

Figure S3

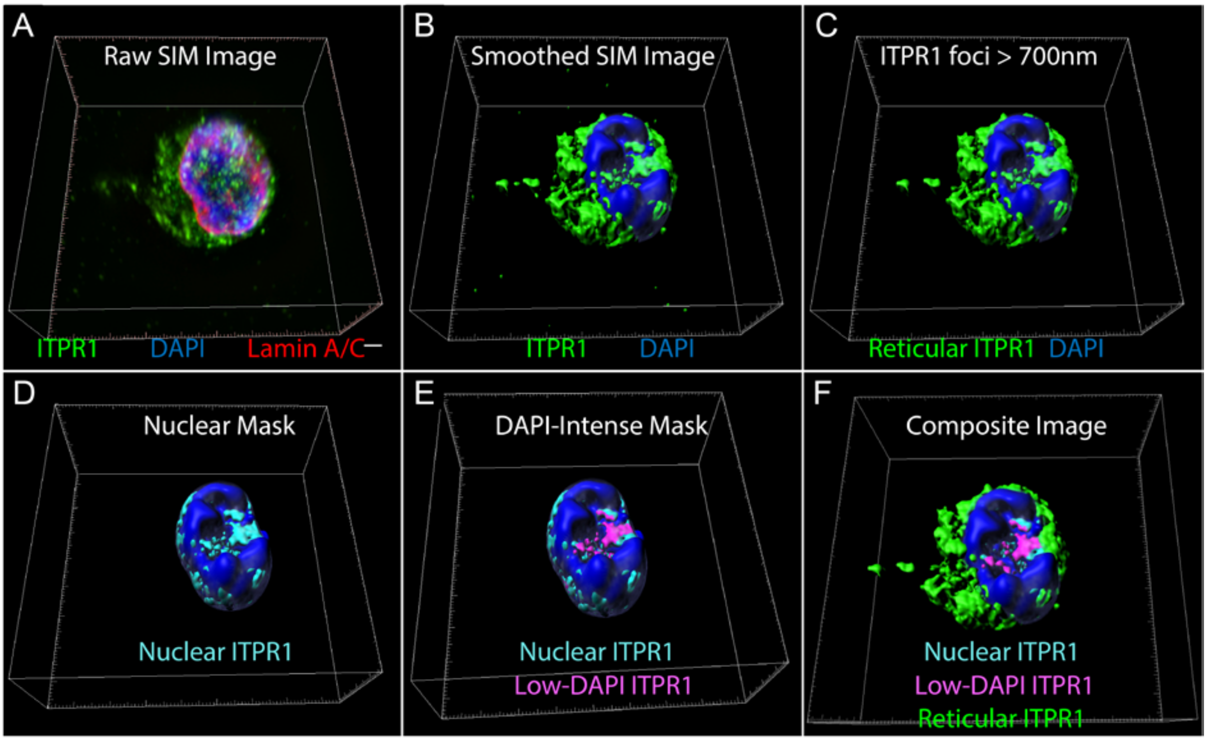

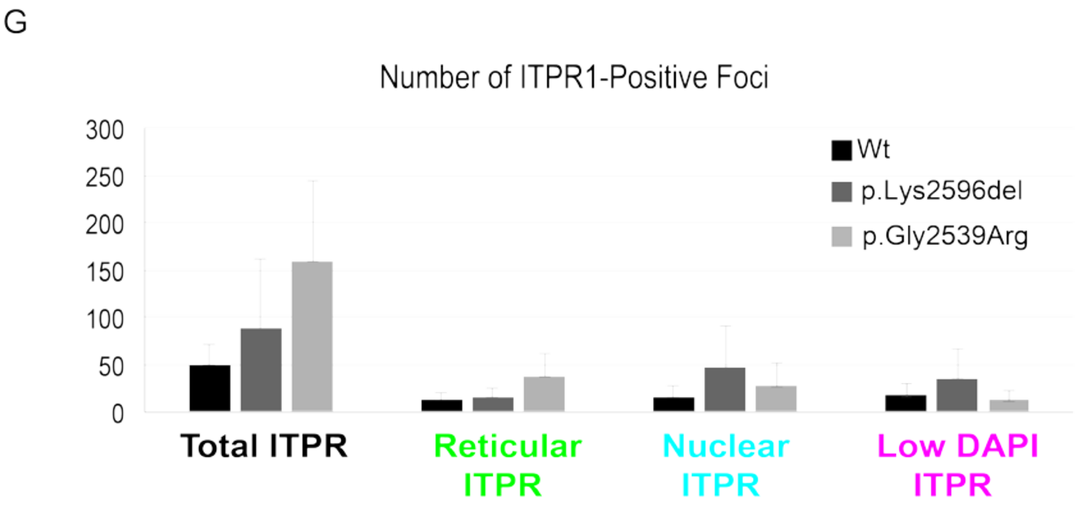

Figure S3: Quantification of ITPR1 levels in Lymphoblastoid cells

Quantification of ITPR1 using Imaris v8.1 (Oxford Instruments). **A.** Raw Structured Illumination Microscopy (SIM) images, Nuceli are labelled using DAPI in blue, ITPR1 in green, Lamin A/C in Red. **B.** Total ITPR1 was measured by smoothing images and selecting ITPR1 above the local background. **C** Reticulated ITPR1 was defined as ITPR1-positive structures larger than 700nm in any direction, ITPR1-positive foci smaller than this were excluded from analysis. **D.** Nuclear ITPR-1 was defined as ITPR1 co-localised with DAPI and was within the LaminA/c stained nuclear envelope. Intranuclear ITPR is shown in cyan. **E.** A second mask high-intensity DAPI staining was generated, (High Dapi Mask). Nuclear

ITPR outwith this mask is shown in magenta. **F.** Composite view of the ITPR1 populations used for quantification; Reticulated ITPR1 (green), Nuclear ITPR1 (cyan), ITPR1 in low DAPI stained areas (Magenta). The nuclear mask is shown in Blue. Scale bar = 1µm. G. Graph of the number of ITPR1-positive immunofluorescent foci using multiple cells from two lymphoblastoid cell lines per genotype . Fluorescent levels are derived from DAPI and secondary antibody conjugates. The primary antibodies were Itpr1 mouse monoclonal antibody (Life Technologies ab166871, 1:100) and Lamin A/C affinity purified goat polyclonal antibody sc-6216 (Santa Cruz Biotechnology, Inc, 1:1000). Structured Illumination Microscopy (SIM) was performed on an Eclipse Ti inverted microscope equipped with a Nikon Plan Apo TIRF 100x objective (NA 1.49, oil immersion) and an Andor DU-897X-5254 camera. Laser lines used were 405nm (blue), 488nm (green) and 640nm (Far Red). Z-step size for Z stacks was set to 0.120 um. For each focal plane, 15 images (5 phases, 3 angles) were captured with the NIS-Elements software. SIM image processing, reconstruction and analysis were carried out using the N-SIM module of the NIS-Element Advanced Research software. In all SIM image reconstructions the Wiener and Apodization filter parameters were kept constant.

Figure S4

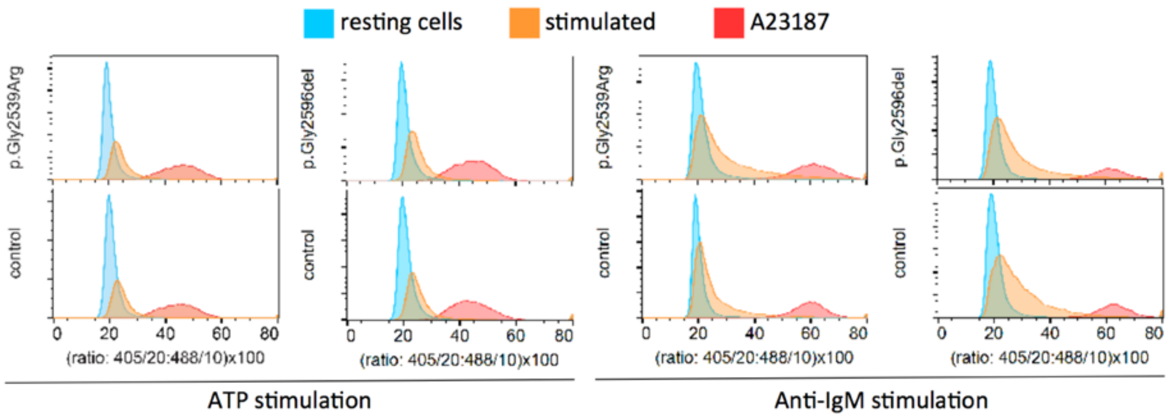

Figure S4: Calcium release in Mutant and Wild-Type LCLs.

To assess intracellular calcium release we used the agonists ATP (10mM) and anti-Human IgM (50µg/ml) added to control and test cell lines. The control cells were labelled with Dil and test cells with Did using the Vybrant kit (Molecular Probes Inc, Eugene, USA). Control and test cell lines were mixed prior to loading with 2µM of the ratiometric calcium indicator Indo-1. Histograms show cell counts over the ratio of Indo-1 fluorescence emission at 405nm and 488nm following excitation with a 355nm laser. In each histogram, populations of untreated cells (light blue), cells treated (orange in both panels) with either 10mM ATP (left panel) or 50µg/ml anti-IgM (right panel) and cells treated with 4µM of the calcium ionophore A23187 (red, positive control) are shown (ref.3, ref.4). Data are representative of all available ITPR1-mutant cell lines of a particular ITPR1 genotype (p.Gly2539Arg or p.Lys2596del). Each cell line was assessed in two independent experiments per agonist. No differences were seen between mutant and control cell lines using either agonist.

References:

1. Singh PP, Affeldt S, Malaguti G, Isambert H 2014, Human dominant disease genes are enriched in paralogs originating from whole genome duplication. PLoS Comput Biol 2014 Jul 31;10(7).

2. Ran FA, Hsu PD, Wright J, Agarwala V, Scott DA, Zhang F. Genome engineering using the CRISPR-Cas9 system. Nat Protoc. 2013 Nov;8(11):2281-308.

3. Lee DH, Park KS, Kong ID, Kim JW, Han BG. Expression of P2 receptors in human B cells and Epstein-Barr virus-transformed lymphoblastoid cell lines. BMC Immunology, 7 (2006), 22.

4. Speck P, Ikeda M, Ikeda A, Lederman HM, Longnecker R. Signal transduction through the B cell antigen receptor is normal in ataxia-telangiectasia B lymphocytes. J Biol Chem. 2002 Feb 8;277(6):4123-7.

Table S1: ITPR1 mutations analysed in this study, and their predicted changes in protein stability.

The recently published cryoelectron microscopy structure of the tetrameric ITPR1 channel (PDB ID: 3JAV) was refined using MODELLER to add missing side chains to the C_α backbone. Twenty FoldX replicates were performed for each pathogenic *ITPR1* mutation (except Val1547Met, which is not present in the structure). Since FoldX does not predict the effects of deletions, we approximated the effects of Lys2596del with a glycine mutation. Although the effects of a deletion can potentially be much different than a glycine mutation, the small predicted effect of the K>G mutation suggests that mutations at this position may be structurally mild.

**Human residue number**[1]	**Mouse residue number**[2]	**Mutation**	**Mutation type**	**Disorder**	**ΔΔG (kcal/mol)**	**Standard deviation (kcal/mol)**
267	267	T>M	*de novo*	infantile-onset SCA	1.00	0.02
267	267	T>R	*de novo*	infantile-onset SCA	1.41	0.11
277	277	S>I	*de novo*	infantile-onset SCA	0.42	0.05
579	594	T>I	*de novo*	infantile-onset SCA	-1.07	0.98
587	602	N>D	inherited and *de novo*	SCA29; ataxic cerebral palsy	-2.07	0.01
1068	1073	P>L	inherited	SCA15	0.61	0.12
1487	1493	S>D	*de novo*	ataxic cerebral palsy	0.15	0.07
1547	N/A	V>M	inherited	SCA29	N/A	N/A
2094	2101	E>Q	*de novo*	Gillespie syndrome	-1.25	0.50
2094	2101	E>G	inherited	Gillespie syndrome	-0.66	0.64
2539	2546	G>R	*de novo*	Gillespie syndrome	5.76	1.91
2596	2603	K>del[3]	*de novo*	Gillespie syndrome	-0.46	0.16

[1]From protein sequence NP_001161744.1 (Q14643-2; ENSP00000306253.8)

[2]From PDB ID: 3JAV

[3]Values given here are for a K>G mutation at this position

Primers Used in Targeted Analysis of ITPR1

Table S2: Primers used in ITPR1 targeted sequencing

oligonucleotide name (total exon number)	Oligonucleotide sequence 5' > 3'
Human	
ITPR1_ex48F	GTAGCGCGACGGCCAGTCCTCCCATGTGCCAGTTG
ITPR1_ex54F	GTAGCGCGACGGCCAGTGTGTGAGATGCTCTCGTTGC
ITPR1_ex55F	GTAGCGCGACGGCCAGTAAACCAAGTTTGCATTATGGG
ITPR1_ex56F	GTAGCGCGACGGCCAGTTTAATCAGCCGTGAATTGGG
ITPR1_ex57F	GTAGCGCGACGGCCAGTGATGGCATTCAGGAAACAGG
ITPR1_ex58F	GTAGCGCGACGGCCAGTCCCAGACTGATCCAGACACC
ITPR1_ex48R	CAGGGCGCAGCGATGACAAGCTCCAGGAAGCAGATCC
ITPR1_ex54R	CAGGGCGCAGCGATGACAGGGTCTGTGATGAGAGAGAGG
ITPR1_ex55R	CAGGGCGCAGCGATGACCGTGTTAGGGAGATACAATGGG
ITPR1_ex56R	CAGGGCGCAGCGATGACTCTTCTTCCAACATCACCTGC
ITPR1_ex57R	CAGGGCGCAGCGATGACTACACTCAACACCGCTGCAT
ITPR1_ex58R	CAGGGCGCAGCGATGACATCACACCCTCGCAGTATCC
Mouse	
m_Itpr1_ex57F	CCCCTCCTGTCTTAACTGTG
m_Itpr1_ex57Ra	CTGCCTGTCCCCACAAGCC

Table S3 Predicted pathogenicity scores for *ITPR1* missense mutations.

gene	g.	c.	p.	ExAC allele count	phenotype	inheritance	PolyPhen2 (score)	SIFT (score)	MutationTaster	Align GVGD
ITPR1	hg19 Chr3:4687357-4687357	800C>T	Thr267Met	0	SCA29, cerebellar ataxia	heterozygous *de novo*	PROBABLY DAMAGING (1.000)	Deleterious (0)	Disease causing (prob value: 0.999)	Class C65 (GV: 0.00 - GD: 81.04)
ITPR1	hg19 Chr3:4687357-4687357	800C>G	Thr267Arg	0	cerebellar ataxia	heterozygous *de novo*	PROBABLY DAMAGING (1.000)	Deleterious (0)	Disease causing (prob value: 0.999)	Class C65 (GV: 0.00 - GD: 70.97)
ITPR1	hg19 Chr3:4687387-4687387	830G>T	Ser277Ile	0	SCA29, cerebellar ataxia	heterozygous *de novo*	PROBABLY DAMAGING (0.999)	Deleterious (0)	Disease causing (prob value: 0.999)	Class C65 (GV: 0.00 - GD: 141.80)
ITPR1	hg19 Chr3:4709128-4709128	1736C>T	Thr579Ile	0	cerebellar ataxia	heterozygous *de novo*	PROBABLY DAMAGING (0.998)	Deleterious (0)	Disease causing (prob value: 0.999)	Class C65 (GV: 0.00 - GD: 89.28)
ITPR1	hg19 Chr3:4709151-4709151	1759A>G	Asn587Asp	0	SCA29; ataxia with ID	heterozygous *de novo*, familial	PROBABLY DAMAGING (0.958)	Deleterious (0)	Disease causing (prob value: 0.999)	Class C15 (GV: 0.00 - GD: 23.01)
ITPR1	hg19 Chr3:4725156-4725156	3203C>T	Pro1068Leu	0	SCA15, adult-onset	heterozygous familial	POSSIBLY DAMAGING (0.792)	Deleterious (0)	Disease causing (prob value: 0.999)	Class C65 (GV: 0.00 - GD: 97.78)
ITPR1	hg19 Chr3:4741593-4741594	4459_4460delinsGA	Ser1487Asp	0	ataxia with ID	heterozygous *de novo*	POSSIBLY DAMAGING (0.905)	Deleterious (0)	Disease causing (prob value: 0.999)	Class C65 (GV: 0.00 - GD: 65.47)
ITPR1	hg19 Chr3:4747877-4747877	4639G>A	Val1547Met	0	SCA29; SCA	heterozygous familial	BENIGN (0.085)	Tolerated (0.41)	Disease causing (prob value: 0.999)	Class C15 (GV: 0.00 - GD: 20.52)

ITPR1	hg19 Chr3:4821267-4821267	6280G>C	Glu2094Gln	0	Gillespie syndrome	heterozygous *de novo*	PROBABLY DAMAGING (0.999)	Deleterious (0.01)	Disease causing (prob value: 1)	Class C25 (GV: 0.00 - GD: 29.27)
ITPR1	hg19 Chr3:4821268-4821268	6281A>G	Glu2094Gly	0	Gillespie syndrome	heterozygous familial	PROBABLY DAMAGING (0.999)	Deleterious (0)	Disease causing (prob value: 1)	Class C65 (GV: 0.00 - GD: 97.85)
ITPR1	hg19 Chr3:4856205-4856205	7615G>C	Gly2539Arg	0	Gillespie syndrome	heterozygous *de novo*	PROBABLY DAMAGING (0.999)	Deleterious (0)	Disease causing (prob value: 1)	Class C65 (GV: 0.00 - GD: 125.13)
ITPR1	hg19 Chr3:4856205-4856205	7615G>A	Gly2539Arg	1 in 120716	Gillespie syndrome	heterozygous *de novo*	PROBABLY DAMAGING (0.999)	Deleterious (0)	Disease causing (prob value: 1)	Class C65 (GV: 0.00 - GD: 125.13)
ITPR1	hg19 Chr3:4856866-4856868	7786_7788delAAG	Lys2596del	0	Gillespie syndrome	heterozygous *de novo*	N/A	N/A	Disease causing (prob value: 0.999)	N/A

Table S3. Predicted pathogenicity scores for ITPR1 missense mutations. All numbering is based on the nucleotide canonical reference sequence NM_001168272.1 (ENST00000302640) and the corresponding protein non-canonical reference sequence NP_001161744.1 (ENSP00000306253.8; Q14643-2), which represent ITPR1 isoform 2 with a total of 2743 amino acids and lacking the 15 amino acid insertion at Asp321 in the ligand transferase domain. All phenotypes are congenital or early onset unless stated otherwise. The pathogenicity predictions were run from the following sites: PolyPhen2 http://genetics.bwh.harvard.edu, SIFT http://grch37.ensembl.org/Homo_sapiens/Tools/VEP/, MutationTaster http://www.mutationtaster.org, and Align GVGD http://agvgd.iarc.fr/agvgd (all run on 8 Feb 2016). SCA, spinocerebellar ataxia; ID, intellectual disability; N/A, not applicable.
